# How accurate is the ‘Surprise Question’ at identifying patients at the end of life? A systematic review and meta-analysis

**DOI:** 10.1186/s12916-017-0907-4

**Published:** 2017-08-02

**Authors:** Nicola White, Nuriye Kupeli, Victoria Vickerstaff, Patrick Stone

**Affiliations:** Marie Curie Palliative Care Research Department, Division of Psychiatry, University College London, 6th Floor, Maple House, 149 Tottenham Court Road, London, W1T 7NF UK

**Keywords:** Surprise question, Accuracy, Prognosis, End of life, Palliative care, Survival

## Abstract

**Background:**

Clinicians are inaccurate at predicting survival. The ‘Surprise Question’ (SQ) is a screening tool that aims to identify people nearing the end of life. Potentially, its routine use could help identify patients who might benefit from palliative care services. The objective was to assess the accuracy of the SQ by time scale, clinician, and speciality.

**Methods:**

Searches were completed on Medline, Embase, CINAHL, AMED, Science Citation Index, Cochrane Database of Systematic Reviews, Cochrane Central Register of Controlled Trials, Open Grey literature (all from inception to November 2016). Studies were included if they reported the SQ and were written in English. Quality was assessed using the Newcastle–Ottawa Scale.

**Results:**

A total of 26 papers were included in the review, of which 22 reported a complete data set. There were 25,718 predictions of survival made in response to the SQ. The c-statistic of the SQ ranged from 0.512 to 0.822. In the meta-analysis, the pooled accuracy level was 74.8% (95% CI 68.6–80.5). There was a negligible difference in timescale of the SQ. Doctors appeared to be more accurate than nurses at recognising people in the last year of life (c-statistic = 0.735 vs. 0.688), and the SQ seemed more accurate in an oncology setting 76.1% (95% CI 69.7–86.3).

**Conclusions:**

There was a wide degree of accuracy, from poor to reasonable, reported across studies using the SQ. Further work investigating how the SQ could be used alongside other prognostic tools to increase the identification of people who would benefit from palliative care is warranted.

**Trial registration:**

PROSPERO CRD42016046564.

**Electronic supplementary material:**

The online version of this article (doi:10.1186/s12916-017-0907-4) contains supplementary material, which is available to authorized users.

## Background

In both the UK and the USA, the Surprise Question (SQ; “Would you be surprised if this patient died within the next × months?”) has been suggested as a trigger for referral to specialist palliative care [1, 2].

It has been estimated that between 69% and 82% of dying patients in the UK would benefit from palliative care input (specialist or generalist) [3]. There were approximately 160,000–170,000 referrals made to specialist palliative care services in the UK between 2008 and 2009 [4]. In the USA, it has been estimated that between 1 million and 1.8 million (7.5% and 8.0%) of hospital admissions have a palliative care need [5].

It has been repeatedly shown that clinicians are inaccurate at prognostication [6–8] and in recognising dying patients [9]. Therefore, there is an increased likelihood that patients who would benefit from palliative care are potentially being missed because validated prognostic tools (e.g. Palliative Prognostic Score [10] or Palliative Prognostic Indicator [11]) are not being used routinely, either due to their perceived complexity or inconvenience [12].

The SQ offers an alternative to a standard prognostic estimate. It does not require clinicians to collect clinical data or to use a scoring algorithm, nor does it require clinicians to make a specific estimate about length of survival; it simply asks whether the respondent would be surprised if the patient were to die within a specified time period (usually the next year). It was originally developed by Joanne Lynn as a method to identify patients who might benefit from palliative care services [13], asking the clinician: “Is this person sick enough that it would be no surprise for the person to die within the next 6 months, or a year?” Since its development, the SQ, or variants thereof, have been incorporated into clinical guidelines such as National Institute for Health and Care Excellence (NICE) for End of Life Care [14], and adopted into routine clinical practice in various settings, including hospitals, hospices, and General Practices. Although developed as a standalone item, it is now included as part of the Gold Standard Framework (GSF) proactive identification guidance tool in the UK [1], in which clinicians are encouraged to ask themselves “Would you be surprised if this patient were to die in the next 6–12 months”. A response of “No” to the SQ may trigger a referral to specialist palliative care services, or to the adoption of a palliative care approach to future care. This parsimonious approach could potentially identify more patients who need palliative care and could be incorporated into routine clinical practice with relative ease and at little or no extra cost.

Yet, how accurate or effective is the SQ at identifying people in the last year of life? Could it be used to identify people who are just days from dying? Is one clinical group more accurate at using the SQ than another? Is the SQ more accurate when used by one professional group rather than another? The present study aims to assess the accuracy of the SQ by time scale, clinician, and speciality.

## Methods

### Data sources and searches

We initially searched the literature using the terms, “surprise question” and “death”. Medline, Embase, CINAHL, AMED, Science Citation Index, Cochrane Database of Systematic Reviews, and Cochrane Central Register of Controlled Trials databases were searched. All databases were searched from inception up to the date of the search (November 2016), including papers still being processed by the databases (for exact search terms, see Table 1). In addition, the references of all included studies were checked and authors of papers were contacted to check for any additional papers and for full papers if only an abstract was identified. After contacting the authors of identified papers, it was discovered that our original search strategy had failed to identify one key paper [15]; therefore, the search terms were amended to include “GSF” as a keyword. The search strategy was then re-run on all databases.

**Table 1:** Search Strategy

Database	Search Terms	# papers search 1 (Aug 2016)	# papers search 2 (Nov 2016)
OVID platform: Medline, Embase, AMED	Dying.mp. Death.mp. mortality.mp. 1 or 2 or 3 (surprise adj1 question).mp. Gsf.mp 5 or 6 4 and 7	55	137
Web of Science	TS = (surprise NEAR/1 question OR GSF) TS = (dying OR death OR mortality) #1 AND #2	31	68
CINAHL (EBSCOhost)	TX surprise question TX GSF TX dying TX mortality TX death 3 OR 4 OR 5 1 OR 2 6 AND 7	13	33
Database of systematic reviews and Cochrane Central Register of Controlled Trials	surprise NEAR/1 question TX GSF TX dying TX mortality TX death 3 OR 4 OR 5 1 OR 2 6 AND 7	8	12
Open Grey	“Surprise Question”	0	0

*GSF* Gold Standard Framework, *TS* topic, *TX* all text

### Study selection

All study designs were included.

### Inclusion

- Studies conducted in human subjects
- Studies reporting the use of the SQ

### Exclusion

- Studies which did not quantify the accuracy of the SQ (or for which this information was not available from study authors)
- Studies that collected data retrospectively
- Not reported in English

Originally, it was planned to exclude all studies that were in abstract form. However, due to the low number of studies initially identified, and the relative low risk associated with including such data, we opted to be inclusive of all studies. We contacted all authors of abstracts to obtain a full study report. If a full study was not available, abstracts from which relevant data could be extracted were included.

### Quality assessment

The quality of studies was assessed with the Newcastle–Ottawa Scale [16]. This scale was selected due to the nature of the studies included (non-randomised controlled trial, observational). The raters met and discussed each domain of the scale, completed one study using the scale, and discussed any discrepancies or difficulties that were identified before completing the assessment on all studies. In order to provide a full account of the accuracy of the SQ, no study was excluded based on the risk of bias score alone; however, if possible, it was planned to undertake a sensitivity analysis to account for the effect of poor quality studies. Each paper was assessed by two individual raters (NW and NK). Any discrepancies were resolved by a meeting of the two reviewers, with a third reviewer (PS) being included if the discrepancy was unresolved.

### Selection

The studies identified from the database searches were screened by two reviewers independently (NW and NK). The first selection criterion was that the abstract/title included the use of the SQ. Any study not meeting this criterion was excluded. At full review, studies were selected for inclusion if they reported a quantifiable measure of the accuracy of the SQ (e.g. the proportion of patients correctly identified as being in the last year of life, as opposed to a qualitative assessment such as “the SQ performed well”). Any discrepancies at either selection point were resolved by a meeting between the two reviewers. If unresolved, a third reviewer (PS) was consulted.

### Extraction

The following data were extracted from each paper:

- A description of the study population (both patient and clinician)
- The format of the SQ that was used in the study (e.g. the length of time to which the SQ related, or whether the SQ referred to expected survival or expected death)
- The accuracy of the SQ (i.e. how many people who were identified as likely to die, did actually die)

### Data synthesis and analysis

A narrative synthesis of the findings from the studies was completed. This included details about the patient population characteristics, clinician characteristics, the format of the SQ, and the outcome (accuracy of SQ).

A quantitative synthesis was completed from those studies where data were available. All authors were contacted if the published data were incomplete or where calculation errors were noted in the published manuscript. Stata v13.0 was used for all analyses.

The accuracy of the SQ was summarised in a 2 × 2 table for each study. The sensitivity (the ability to recognise those who were dying), specificity (the ability to recognise those who were not dying), positive predictive value (PPV, the proportion of patients who died when the clinician predicted dying), and the negative predictive value (NPV, the proportion of patients who survived when the clinician predicted survival) were calculated [17]. The area under the curve (AUROC), also known as the c-statistic value, was calculated. This statistic compares the number of correct estimates (sensitivity) against the number of false estimates (1-specificity). A score of 0.5 indicates a model with poor predictive value, meaning that clinicians are no better than chance at identifying a person nearing the end of their life. An increase in the value (to a maximum score of 1) indicates an increase in the level of clinician accuracy.

The accuracy overall, by timeframe, by profession, and by speciality, was calculated by meta-analysis, using the “*metaprop*” command in Stata. These data were used to assess heterogeneity of the data synthesis using the *I* ^2^ statistic. Where possible, a sub-analysis of accuracy by clinician profession, patient group, and clinical setting were completed. To account for the 0% and 100% limits, the data obtained were transformed using the Freeman Tukey double arcsine method, and a meta-analysis was completed using the DerSimonian–Laird method with inverse variance weighting and then back transformed to present the percentage accuracy. We examined the impact of the time-frame of the SQ on its diagnostic accuracy. For this analysis, the time frame was categorised into up to 30 days, up to 6 months, and up to 12 months.

Publication bias was assessed using a funnel plot (Additional file 1: Figure S1).

### Role of the funding source

This review was completed as part of a PhD studentship awarded from University College London. The funders had no role in this systematic review.

## Results

**Fig. 1:**
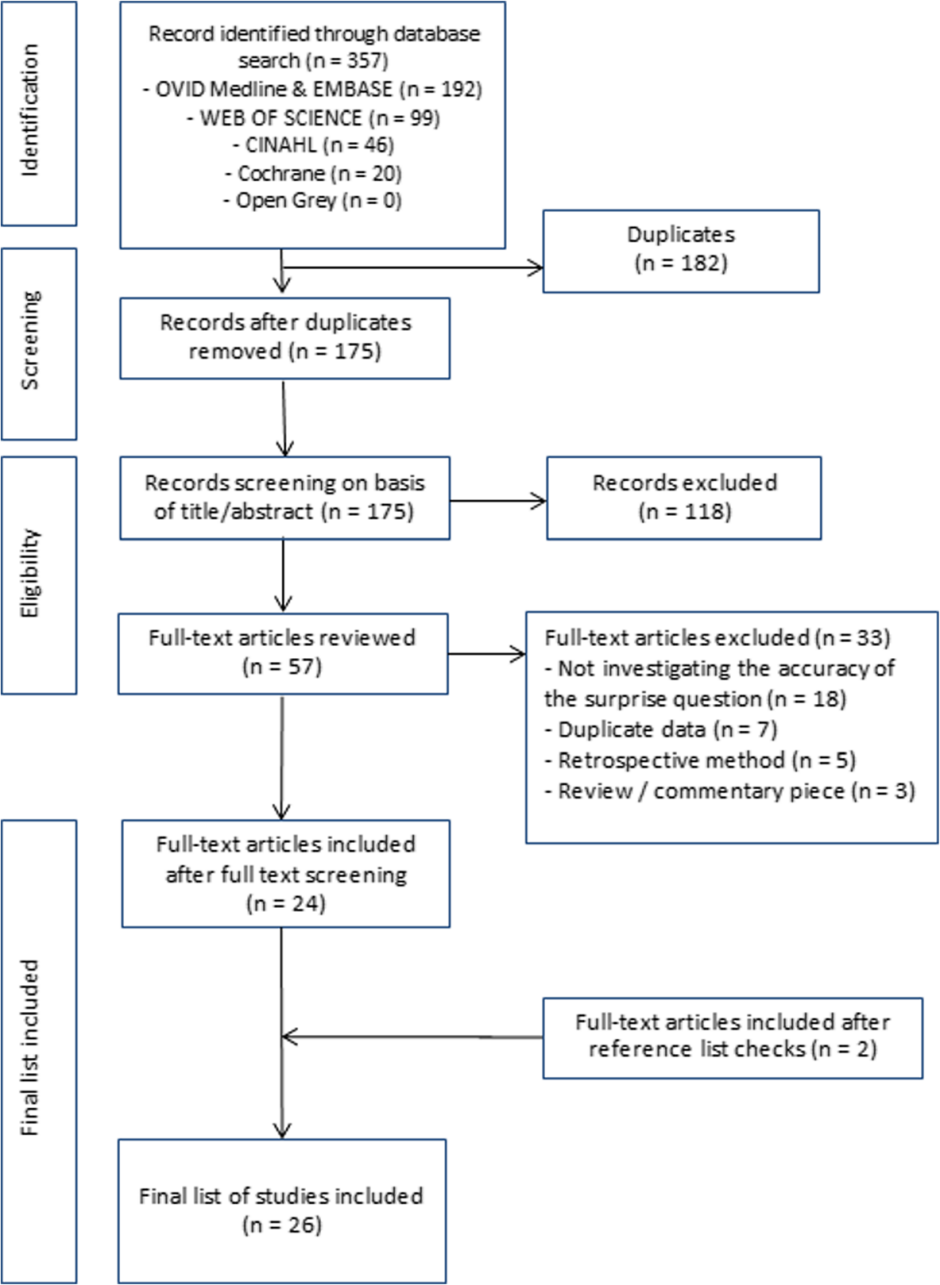
PRISMA flowchart

In total, 357 studies were identified from the database searches (Fig. 1). No studies were identified through the grey literature search. Of those studies initially identified, 182 were subsequently found to be duplicates. There were 175 studies that were screened by title and abstract, of which 118 were excluded. Of the 57 full text articles retrieved, 34 were excluded for various reasons (Additional file 2: Table S1). Three additional studies were identified from a search of the references of the included studies. In total, 26 papers were included in this review [15, 18–42].

We were able to obtain data from one unpublished paper with which we replaced one of the abstracts [33]. Another abstract [41] was excluded because the full paper had also been published [43].

Each paper included underwent a quality assessment with high agreement between the two raters (ICC 0.93) (Additional file 3: Table S2). No paper was excluded on the basis of their quality score.

A summary of the studies included can be seen in Table 2. Of the 26 papers, ten (38%) were from the UK and nine (35%) were from the USA. Ten (38%) of the studies were presented in abstract form, the rest were full papers. Eight of the included studies related to patients with end-stage renal disease, six related to cancer patients, four related to patients with heart failure, one study related to patients with sepsis, one study related to chronic obstructive pulmonary disease and six studies included patients with a variety of different diagnoses. The majority of studies (15, 58%) specified 12 months as the relevant period for the SQ. Eight papers specified a time frame of 6 to 12 months. Three papers specified shorter time periods.

**Table 2:** Detail of the studies included in the review

First author	Year	Country	Time frame of SQ	Diagnosis	Clinician	Location/setting	Total	Patient age (Mean, SD)	Patient Sex (M:F)	Mean QS (/ 9)
Amro [18]	2016	USA	12 months	End-stage renal failure	Nephrologists	Dialysis unit/hospital	201	66	105:46	6.5
Barnes [35]	2008	UK	12 months	Heart failure	General practitioners	General practice	231	77 (71–82)^c^	120:111	4.5^h^
Carmen^a^ [19]	2016	Spain	12 months	End stage renal failure	Medical staff	HD unit	49	NR	NR	5.0^h^
Cohen [20]	2010	USA	6 months	End-stage renal failure	Nephrologists	HD 5 units	450^b^	61 (17)	285:225	8.0
Da Silva [40]	2013	UK	12 months	End-stage renal failure	Nurses and Nephrologists	HD units	344	63.6 (15.5)	221:123	6.5
Fenning [30]	2012	UK	6–12 months	Heart failure	Clinical team	Hospital cardiology unit	172	66	105:67	9.0
Feyi [21]	2015	UK	6–12 months	End-stage renal failure	Consultant renal physician, consultant in palliative medicine and renal nursing staff	Dialysis unit/hospital	178	NR	48:22	6.0
Gardiner [36]	2013	UK	12 months	Any diagnosis	Doctor (Nursing Staff)	Acute hospital	297	78^d^	136:161	5.5
Reid^a^ [43]	2012	UK	during this admission	Any diagnosis	Nursing staff	5 wards and 2 specialist palliative care beds	6703	80.6 (11.2)	20:40 (partial)	5.0^h^
Gopinathan^a^ [31]	2016	India	6 months	End-stage renal failure	Principal investigator	Tertiary care hospital	39	NR	NR	5.0^h^
Haga [15]	2012	UK	6–12 months	Heart failure	Specialist heart failure nurse	Hospital community-based patients	138	77 (10)	91:47	7.5
Halbe^a^ [22]	2015	Germany	12 months	Cancer	Medical staff	Haematology and oncology outpatient clinic	651	NR	NR	5.5
Hamano [23]	2015	Japan	7 days	Cancer	Palliative care physicians’	16 palliative care units, 19 hospital-based palliative care teams, and 23 home-based palliative care services	2361	69.1 (12.6)	1358:1003	6.5
Johnson [37]	2012	UK	12 months	Heart failure	Heart failure nurse	Cardiology palliative care team	126	78 (10.7)^e^	78:47	5.5
Khan^a^ [44]	2014	USA	6 months	Any diagnosis	Intensivists	Medical intensive care unit	500	NR	NR	3.0^h^
Lefkowits^a^ [38]	2015	USA	12 months	Cancer	Physicians, advanced practice providers, 4 nurses	Academic institution	263	NR	0:263	5.5
Lilley [39]	2016	USA	12 month	Any diagnosis	Surgeons	Tertiary care academic hospital	163	NR	78:85	7.5
Moroni [25]	2014	Italy	12 months	Cancer	General practitioners	GPs	231	70.2 (0.9)	117:114	8.5
Moss [27]	2008	USA	12 months	End-stage renal failure	Nurse practitioner	3 HD units	147	66.4 (14.8)	NR	7.5
Moss [26]	2010	USA	12 months	Cancer	Oncologists	Academic cancer centre	826	60 (13)	126:727	7.5
O'Callaghan [28]	2014	New Zealand	6 months	Any diagnosis	Two expert palliative care clinicians (doctor and a nurse)	Tertiary New Zealand teaching hospital	501	NR	47:50^g^	7.0
Pang [29]	2013	Hong Kong	12 months	End-stage renal failure	Nephrologists	Dialysis centre	367	60.2 (12.3)	204:163	6.5
South^a^ [32]	2011	UK	6–12 months	COPD	Clinician	Nurse-led unit for COPD	199	70 (37–93)^f^	92:107	4.5^h^
Strout^a^ [42]	2016	USA	30 days	Sepsis	Emergency physicians	Emergency Dept	330	NR	181:149	4.5^h^
Thiagarajan [33]	2012	UK	12 months	Any diagnosis	Doctor, nurse, physiotherapist and occupational therapist	Inpatient of an acute/geriatric medical ward	130	80.7 (18–104)^f^	55:75	7.5
Vick^a^ [34]	2015	USA	12 months	Cancer	Oncology clinicians	Cancer centre	4617	NR	NR	3.0^h^

^a^Abstract

^b^Out of a total sample of 512, 450 had a response to the SQ

^c^Median (IQR)

^d^Median

^e^Mean number of days (IQR)

^f^Range

^g^Included one transgender and one unknown

^h^Excluded for sensitivity analysis

*COPD* chronic obstructive pulmonary disease, *NR* not reported

We were able to extract data from 22/26 papers either directly from the paper or after contacting the author (Table 3).

**Table 3:** Individual study diagnostic test results

First author	SQ time frame	Total (n)	SQ responses				Diagnostic test results				
			SQ	n	Died (n)	Survived (n)	Sensitivity	Specificity	PPV	NPV	c-statistic
							% (95% CI)				
SQ up to 12 months											
Amro [18]	12 months	201	No	50	22	28	56.4	82.7	44	88.7	0.696
			Yes	151	17	134	(39.6–72.2)	(76–88.2)	(30–58.7)	(82.6–93.3)	(0.612–0.78)
Carmen [19]	12 months	49	No	20	7	13	77.8	67.5	35	93.1	0.726
			Yes	29	2	27	(40–97.2)	(50.9–81.4)	(15.4–59.2)	(77.2–99.2)	(0.565–0.888)
Feyi [21]	6–12 months	178	No	58	37	21	72.5	83.5	63.8	88.3	0.78
			Yes	120	14	106	(58.3–84.1)	(75.8–89.5)	(50.1–76)	(81.2–93.5)	(0.71–0.85)
Halbe [22]	12 months	651	No	139	71	68	65.7	87.5	51.1	92.8	0.766
			Yes	512	37	475	(56–74.6)	(84.4–90.1)	(42.5–59.6)	(90.2–94.9)	(0.719–0.813)
Moroni [25]	12 months	231	No	126	87	39	83.7	69.3	69	83.8	0.765
			Yes	105	17	88	(75.1–90.2)	(60.5–77.2)	(60.2–77)	(75.3–90.3)	(0.711–0.819)
Moss [26]	12 months	147	No	34	10	24	45.5	80.8	29.4	89.4	0.631
			Yes	113	12	101	(24.4–67.8)	(72.8–87.3)	(15.1–47.5)	(82.2–94.4)	(0.519–0.743)
Moss [27]	12 months	826	No	131	53	78	74.6	89.7	40.5	97.4	0.822
			Yes	695	18	677	(62.9–84.2)	(87.3–91.7)	(32–49.4)	(95.9–98.5)	(0.769–0.874)
O'Callaghan [28]	12 months	501	No	99	67	32	62.6	91.9	67.7	90	0.772
			Yes	402	40	362	(52.7–71.8)	(88.7–94.4)	(57.5–76.7)	(86.7–92.8)	(0.724–0.82)
Pang [29]	12 months	367	No	109	27	82	61.4	74.6	24.8	93.4	0.68
			Yes	258	17	241	(45.5–75.6)	(69.5–79.3)	(17–34)	(89.7–96.1)	(0.603–0.756)
Thiagarajan [33]	12 months	130	No	83	47	36	75.8	47.1	56.6	68.1	0.614
			Yes	47	15	32	(63.3–85.8)	(34.8–59.6)	(45.3–67.5)	(52.9–80.9)	(0.534–0.695)
Vick [34]	12 months	4617	No	796	374	422	58.3	89.4	47	93	0.739
			Yes	3821	267	3554	(54.4–62.2)	(88.4–90.3)	(43.5–50.5)	(92.2–93.8)	(0.719–0.758)
Fenning [30]	6–12 months	172	No	38	6	32	35.3	79.4	15.8	91.8	0.573
			Yes	134	11	123	(14.2–61.7)	(72.1–85.4)	(6.02–31.3)	(85.8–95.8)	(.452,– .695)
Barnes [35]	12 months	231	No	14	11	3	11.6	97.8	78.6	61.3	0.547
			Yes	217	84	133	(5.92–19.8)	(93.7–99.5)	(49.2–95.3)	(54.5–67.8)	(0.512–0.581)
South [32]	6–12 months	199	No	96	14	82	93.3	55.4	14.6	99	0.744
			Yes	103	1	102	(68.1–99.8)	(47.9–62.7)	(8.21–23.3)	(94.7–100)	(0.669–0.818)
Haga [15]	6–12 months	138	No	120	39	81	88.6	13.8	32.5	72.2	0.512
			Yes	18	5	13	(75.4–96.2)	(7.57–22.5)	(24.2–41.7)	(46.5–90.3)	(0.453–0.571)
Da Silva [40]	12 months	3896	No	938	281	657	49.6	80.3	30	90.4	0.65
			Yes	2958	285	2673	(45.5–53.8)	(78.9–81.6)	(27–33)	(89.2–91.4)	(0.628–0.671)
SQ up to 6 months											
Cohen [20]	6 months	450	No	71	39	32	37.9	90.8	54.9	83.1	0.643
			Yes	379	64	315	(28.5–48)	(87.2–93.6)	(42.7–66.8)	(79–86.7)	(0.594–0.693)
Khan [44]	6 months	500	No	238	148	90	82.2	71.9	62.2	87.8	0.77
			Yes	262	32	230	(75.8–87.5)	(66.6–76.7)	(55.7–68.4)	(83.2–91.5)	(0.733–0.808)
O'Callaghan [28]	6 months	501	No	99	56	43	72.7	89.9	56.6	94.8	0.813
			Yes	402	21	381	(61.4–82.3)	(86.6–92.6)	(46.2–66.5)	(92.1–96.7)	(0.761–0.865)
Gopinathan [31]	6 months	39	No	19	5	14	83.3	57.6	26.3	95	0.705
			Yes	20	1	19	(35.9–99.6)	(39.2–74.5)	(9.15–51.2)	(75.1–99.9)	(0.52–0.889)
SQ imminent											
Gibbins [41]	Admission	6642	No	327	215	112	57	98.2	65.7	97.4	0.776
			Yes	6315	162	6153	(51.9–62.1)	(97.9–98.5)	(60.3–70.9)	(97–97.8)	(0.751–0.801)
Hamano [23]	7 days	2361	No	931	282	649	84.7	68	30.3	96.4	0.763
			Yes	1430	51	1379	(80.4–88.4)	(65.9–70)	(27.4–33.4)	(95.3–97.3)	(0.742–0.785)
Hamano [23]	30 days	2361	No	1851	1066	785	95.6	37	57.6	90.4	0.663
			Yes	510	49	461	(94.2–96.7)	(34.3–39.7)	(55.3–59.9)	(87.5–92.8)	(0.648–0.678)
Strout [42]	30 days	330	No	108	15	93	48.4	68.9	13.9	92.8	0.586
			Yes	222	16	206	(30.2–66.9)	(63.3–74.1)	(7.99–21.9)	(88.6–95.8)	(0.493–0.68)

Sensitivity (the ability to recognise those who were dying, e.g. 15/31 for study by Strout [42])

Specificity (the ability to recognise those who were not dying, e.g. 206/299 for study by Strout [42])

*PPV* positive predictive value (the proportion of patients who died when the clinician predicted dying, e.g. 15/108 for study by Strout [42]), *NPV* negative predictive value (the proportion of patients who survived when the clinician predicted survival, e.g. 206/222 for study by Strout [42])

## Accuracy of the SQ

The outcomes of 25,718 estimates were reported in the 22 studies with complete data. Patients died within the specified timeframe (whatever it was in that particular study) on 4217 occasions (16%). A response of “No, I would not be surprised” was given on 6495 occasions (25%) and clinicians’ intuitions were ‘correct’ (about whether they should or should not be surprised) in 20,964/25,718 cases (82%). Most of the correct attributions occurred when clinicians indicated that they would be surprised if the patient died within the specified time period (19,223 occasions) and they did in fact survive for that length of time (17,985 occasions).

The results across the studies (Table 3) showed a wide variation in the reported accuracy of the SQ. The sensitivity ranged between 11.6% and 95.6% and a range of 13.8% to 98.2% was reported for specificity. The PPV ranged from 13.9% to 78.6%, and the NPV ranged from 61.3% to 99%. The AUROC score (c-statistic) across the studies ranged from 0.512 to 0.822.

There was no indication of publication bias from the funnel plot (Additional file 1: Figure S1).

**Fig. 2:**
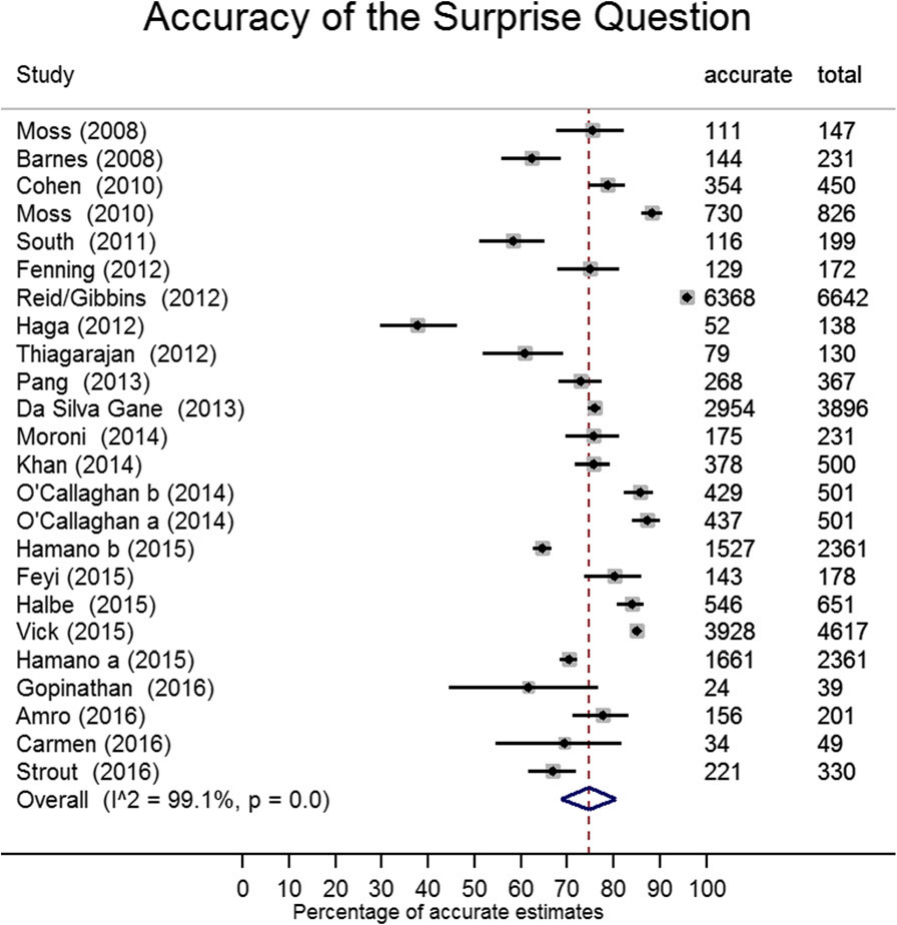
Proportionate data of the number of correct estimates out of the total number of responses

**Fig. 3:**
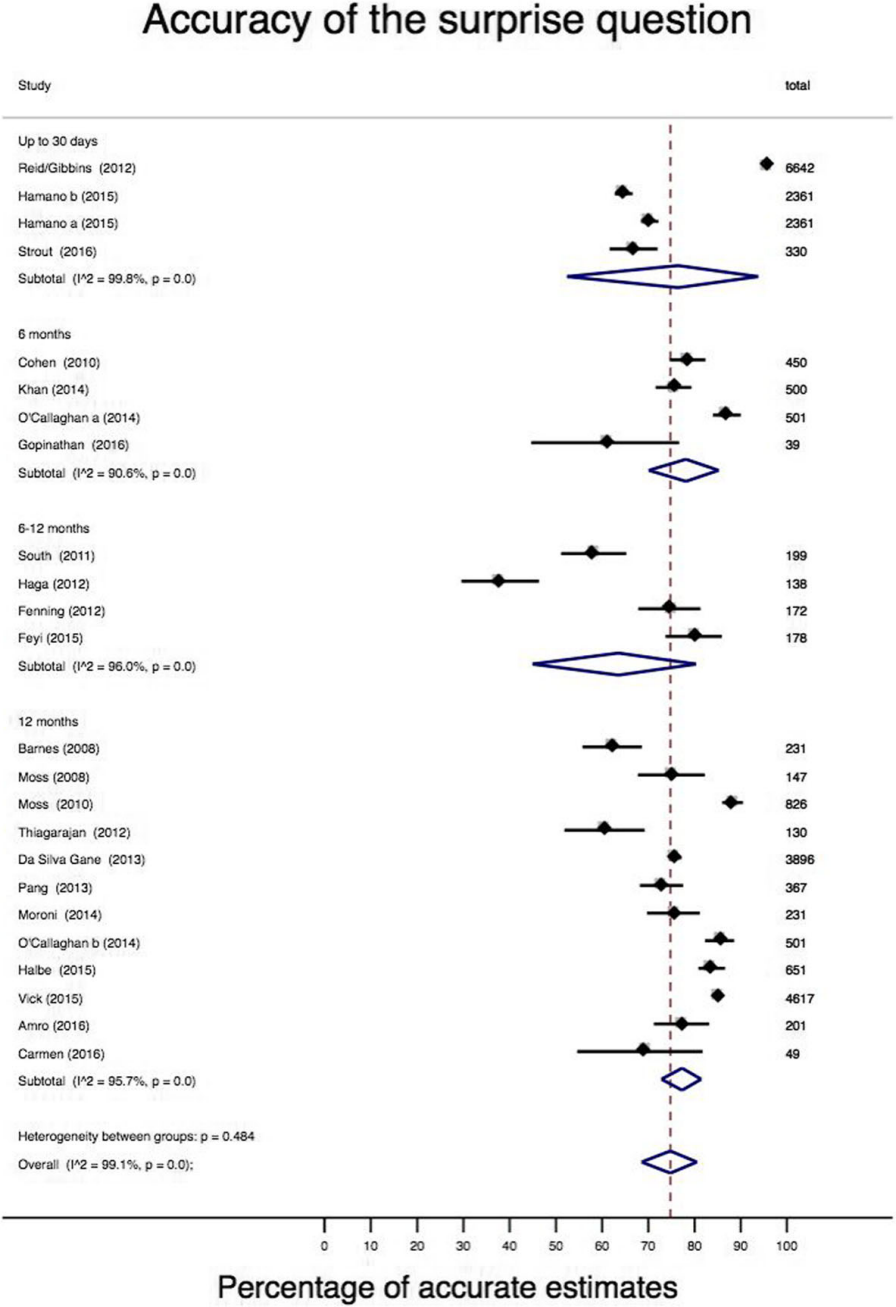
Meta-analysis of the accuracy of the surprise question by time frame

On meta-analysis, the pooled level of accuracy, that is the number of times the clinician correctly predicted the outcome of a patient, was 74.8% (95% CI 68.6–80.5; *I* ^2^ = 99.1%, 95% CI 99–99). The studies were sorted by date of publication and there appeared to be no trend by year (Fig. 2). After a sensitivity analysis for lower quality rated scores, in which eight papers were removed [19, 31, 32, 34, 35, 41, 42, 44], the pooled accuracy level increased to 75.4% (95% CI 70.8–79.7; *I* ^2^ = 96.8, 95% CI 96–98). Those studies that used a shorter time frame for the SQ (up to 6 months) had a pooled estimate of 76.6% (95% CI 61.6–88.8%; *I* ^2^ 99.6%, 95% CI 100–100), and when the time frame was reduced to imminent death (i.e. 7 days, 30 days or ‘this admission’) the pooled accuracy estimate was 76.4% (95% CI 52.4–93.8; *I* ^2^ 99.8%, 95% CI 100–100) (Fig. 3). The meta-regression indicated that the increase in time frame did not impact on the diagnostic accuracy of the SQ: comparing up to 30 days with 12 months (difference in accuracy = 0.8%, 95% CI –12.8 to 14.5, *P* = 0.901) and comparing up to 6 months with 12 months (difference = 4.3%, 95% CI –10.8 to 19.4, *P* = 0.561).

One unpublished paper [33] reported the results of the SQ and a modified version of the SQ, by asking clinicians “would you be surprised if this person was to be alive in a year’s time?” By rewording the question, they found that specificity was improved (i.e. correctly identifying those who do not die) but sensitivity was reduced (i.e. less correct predictions about those who will die).

## The SQ by profession and by specialty

One paper reported the difference in performance of the SQ when used by nurses and doctors [40]. As a result of additional data supplied by the author, it was possible to calculate the sensitivity, specificity, PPV, and NPV for both doctors and nurses (Table 4).

**Table 4:** Surprise Question accuracy by profession

Total		Died	Survived	Sensitivity	Specificity	PPV	NPV	c-statistic
Doctors								
No	218	71 (33)	147 (67)					
Yes	442	26 (6)	416 (94)	73	74	33	94	0.735
Total	660	97 (15)	563 (85)	(63–82)	(70–78)	(26–39)	(92–96)	(0.688–0.783)
Nurses								
No	720	210 (29)	510 (71)					
Yes	2516	259 (10)	2257 (90)	45	82	29	90	0.632
Total	3236	469 (14)	2767 (86)	(40–49)	(80–83)	(26–33)	(86–91)	(0.608–0.655)

Sensitivity (the ability to recognise those who were dying, e.g. 71/97 for doctors)

Specificity (the ability to recognise those who were not dying, e.g. 26/97 for doctors)

*PPV* positive predictive value, the proportion of patients who died when the clinician predicted dying, e.g. 71/218 for doctors), *NPV* negative predictive value, the proportion of patients who survived when the clinician predicted survival, e.g. 416/442 for doctors)

Doctors appeared to be better at predicting dying within 12 months, with a sensitivity of 73% (95% CI 63–82) and specificity of 74% (95% CI 70–78) compared to a sensitivity of 45% (95% CI 40–49) and specificity of 82% (96% CI 80–83) for nurses. The c-statistic for doctors was 0.735 (95% CI 0.688–0.783) compared to 0.632 (95% CI 0.608–0.655) for nurses.

Of the 22 papers that included data, eight reported research conducted within a haemodialysis context [18–21, 27, 29, 31, 40], five were within oncology [22, 23, 25, 26, 34], and nine papers were on other areas (five on all diagnoses [28, 33, 35, 41, 44], one on sepsis [42], two on heart disease [15, 30], and one on COPD [32]). Table 5 highlights the range of scores from the individual studies reporting from each specialty.

**Table 5:** Diagnostic scores of the accuracy of the Surprise Question across specialties

	Studies	Estimates	PPV	NPV	Sensitivity	Specificity	c-statistic
	(n)	(n)	Mean (SD), Range	Mean (SD), Range	Mean (SD), Range	Mean (SD), Range	Mean (SD), Range
Oncology	6	11,047	49.3 (13.5), 30.3–69	92.3 (4.9), 83.8–97.4	77.1 (13.7), 58.3–95.6	73.5 (20.5), 37–89.7	0.753 (0.052), 0.663–0.822
Renal	8	5327	38.5 (14.4), 24.8–63.8	90.2 (3.8), 83.1–95	60.6 (16.2), 37.9–83.3	77.2 (10.4), 57.6–90.8	0.689 (0.049), 0.631–0.78
Other	10	9344	46.4 (24.5), 13.9–78.6	85.5 (13.3), 61.3– 99	62.8 (25.5), 11.6–93.3	71.4 (26.7), 13.8–98.2	0.671 (0.114), 0.512–0.822

*PPV* positive predictive value, *NPV* negative predictive value

**Fig. 4:**
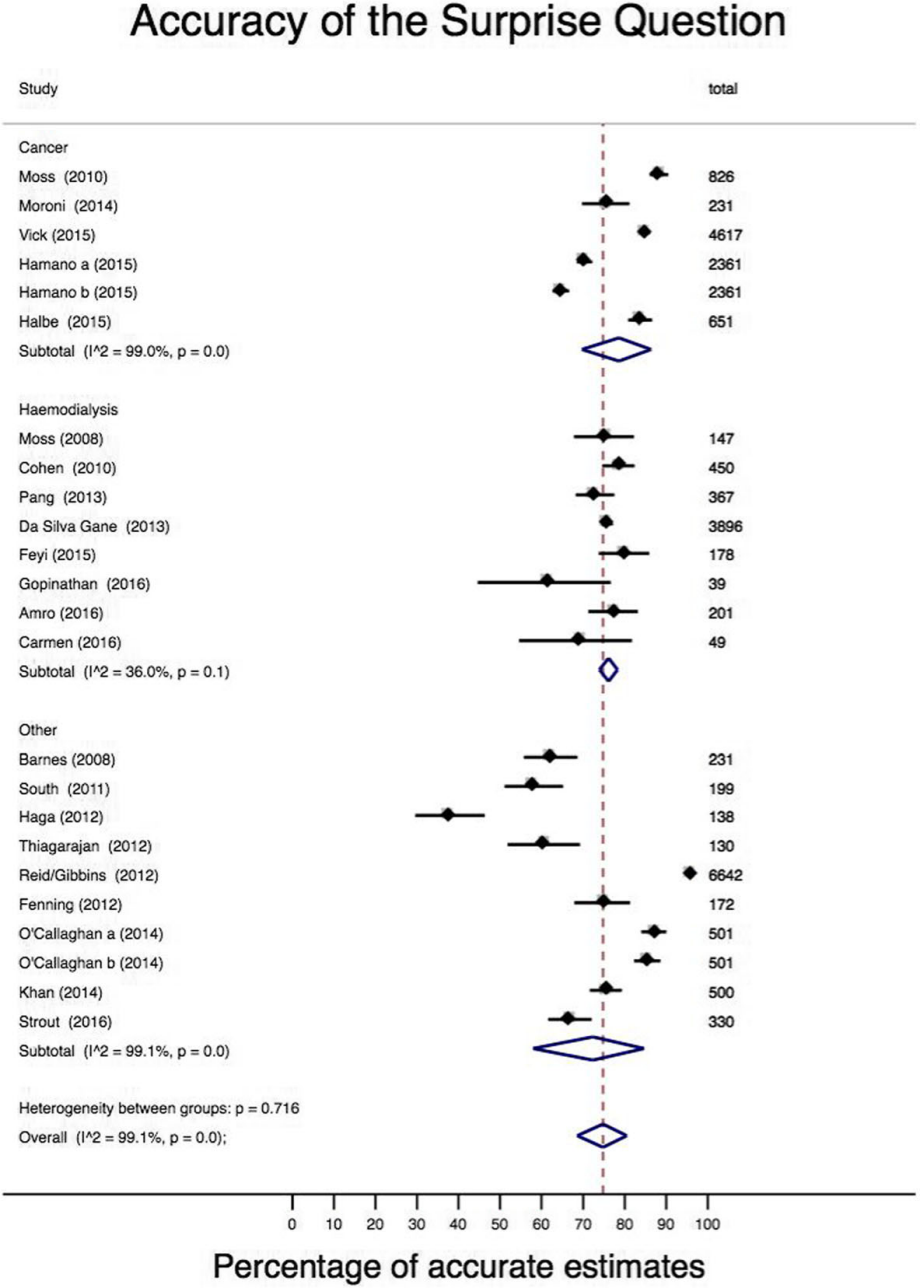
Meta-analysis of the accuracy of the surprise question by specialty

On meta-analysis, the pooled accuracy for oncology was 78.6% (95% CI 69.7–86.3; *I* ^2^ = 99.0%, 95% CI 99–99). The pooled accuracy for renal was 76.1% (95% CI 73.9–78.3%; *I* ^2^ = 36.0%, 95% CI 0–72). The pooled accuracy estimate for the other group was 72.3% (95% CI 58.0–84.6; *I* ^2^ = 99.1%, 95% CI 99–99) (Fig. 4).

## Studies with incomplete data

Four studies had insufficient data to be included in the meta-analysis. Three authors responded to requests, but were unable to provide additional data to that presented in the paper [37, 39]. One author was not contactable [38].

One study [37] did not report data on the accuracy of a ‘Yes’ response to the SQ. In this study, it was reported that specialist heart failure nurses would not be surprised if 88/126 patients would have died within 12 months. In fact, 78/88 (89%) of those patients did die within 12 months. However, no data were reported about the outcomes for the 38 patients where the heart failure nurses would have been surprised if the patient had died. One study only contained data relating to the responses from the clinicians but not the outcome [36]. Gardiner et al. (2013) described the responses from doctors and nurses for two survival time points: 12 months and “*death during this admission*”. Of 297 patients, doctors would not have been surprised if 123 had died within 12 months, or if 50 had died during the admission to hospital. Out of a total of 473 patients, nurses predicted that 180 would die within the next 12 months, and 74 would die during the admission. The actual survival figures were not reported [36]. Lilley et al. [39] reported on the accuracy of 28 clinicians who provided responses for 163 patients. Their results show a ‘No’ SQ response was given in 93 cases (60%). They reported a sensitivity of 81% (95% CI 71–91%), a specificity of 51% (41–61%), a PPV of 52% (42–61%), and a NPV of 82% (72–91%). The exact breakdown of outcome by SQ prediction was not presented [39]. Lefkowits et al. [38] report the accuracy of the SQ within gynaecological oncology and across physicians. They asked 22 clinicians (18 gynaecologic oncology physicians and advanced practice providers (APP); four chemotherapy nurses) the SQ with a timeframe of 12 months. They reported the unadjusted odds ratio for death within a year associated with a ‘No’ SQ response; physicians 5.6 (*P* < 0.001), APP 9.2 (*P* < 0.001) and nurse 6.9 (*P* < 0.001). They reported that the APP group had the highest sensitivity (79.5%) and the nurses had the best specificity (75.6%). No further data was presented [38].

## Discussion

The reported accuracy of the SQ varied considerably between studies. The c-statistic ranged from 0.512 (poor) to 0.822 (good). The PPV ranged from 13.9% to 78.6%. This degree of heterogeneity was not uncommon in studies assessing the accuracy of clinicians’ prognostic estimates [6].

On meta-analysis, the overall accuracy of the SQ was approximately 75%. However, this overall estimate should be reviewed with caution given the low proportion of people who died within each study (16%) and the low number of high quality studies included (*I* ^2^ = 99%). There was virtually no difference in level of accuracy when considering studies in which the timeframe of the SQ had been reduced, which suggests that even when the patient is thought to be imminently dying, there is only moderate accuracy and continued uncertainty. A major limitation of the meta-regression analysis completed in this review was the lack of power due to the small sample size (n = 24). Therefore, a significant difference between time frames of the SQ was less likely to be observed.

One study presented data about the differences in accuracy between different professional groups at using the SQ. This suggested that doctors’ responses to the SQ (c-statistic 0.735) may be more accurate than nurses’ (c-statistic 0.632); however, more research is needed to fully address this question.

The variation in the accuracy of the SQ might be due as much to variations in disease trajectory in the last year of life as it is due to variations in the prognostic ability of the clinicians. There is some evidence that the SQ may be slightly better when used in oncology patients rather than in renal or other disease groups. The pooled accuracy for oncology was 79% compared to 76% for renal and 72% for other disease groups. This supports the idea that patients with a cancer diagnosis have a more predictable disease trajectory [45]. However, there was little variation between the disease groups and so these data should be interpreted cautiously. Another recent review on the SQ (using different inclusion and exclusion criteria to our own) [46] also found that accuracy was slightly better in oncology patients than in other disease groups.

When proposing the original definition of the SQ, Lynn suggested that the accuracy of the outcome was not actually that relevant as all patients identified by this question would typically need the services of palliative care such as advance care planning, home care assistance or financial support [13]. However, it is often the case that clinicians delay referring to palliative care services because they feel that the judgment should be made on the basis of a reasonably accurate and relatively short prognosis. This review highlights that, intuitively, clinicians are actually quite good at excluding patients with longer survival times but that use of the SQ alone is likely to lead to identification of a substantial number of patients who are not necessarily approaching the ends of their lives. However, the review could not provide evidence about how many patients who were identified (or missed) by the SQ actually had palliative care needs.

What is apparent from the data presented is that using the SQ to identify patients with a limited prognosis will detect at least as many ‘false positives’ as ‘true positives’. In most circumstances, the consequences of misclassification by the SQ are rarely likely to be clinically important. For instance, erroneously including patients on a palliative care register who are not actually in the last year of life is unlikely to adversely affect their care, and indeed may result in better provision of holistic care. However, the recognition that half of patients included on such registers, as a result of the SQ, may not actually be in the last year of life has resource implications (e.g. additional staff time, care planning and multi-disciplinary involvement). It is thus not clear whether the SQ is a cost-effective way of identifying patients potentially suitable for palliative care. A careful balance is needed between identifying more people with unmet palliative care needs in a timely way while not over-burdening limited resources with too many patients in need of good care for long-term conditions over a much longer period.

It should be acknowledged that the SQ is usually used as part of a wider prognostic assessment that includes both general measures of performance status and disease burden along with disease-specific indicators [1, 2]. It is possible that the combination of the SQ with these other prognostic measures may well be more accurate than the SQ used in isolation. This, however, was not the focus of this systematic review and further work is needed in order to evaluate the accuracy of these approaches and to determine whether other prognostic tools (e.g. Prognosis in Palliative Care Study [47], Palliative Prognostic Score [10] or Palliative Performance Scale [48]) would be a more accurate way of identifying patients approaching the end of life, or whether accuracy could be further improved by using the SQ alongside other tools such as the Palliative Outcome Scale, which can identify and document changes in the patient’s condition over time [49].

Our study had a number of strengths. This was the first systematic review of the SQ that has attempted a meta-analysis of all studies reporting data on the SQ, including shorter time scales such as 7 days. We adopted a broad search strategy to identify any potentially relevant papers. We also obtained missing data by contacting relevant authors and requesting unpublished results. We have also appraised the quality of the papers included in our study and have conducted a sensitivity analysis to determine whether our conclusions are robust. Finally, each stage of the review process was undertaken by two independent reviewers to ensure rigor.

Our study had a number of limitations. It was difficult to devise a search strategy to capture all of the relevant papers. There is no agreed methodology for conducting a search of the literature for this type of research question. It is therefore possible that some papers may have been missed. In order to perform a meta-analysis of data, we combined studies that had evaluated the accuracy of the SQ over different time-frames, in widely differing patient groups and with different groups of clinicians. It may be argued that this is not a legitimate approach in terms of clinical heterogeneity as the performance of the SQ may be very different in each of these different circumstances. Further work is clearly needed to investigate any such differences (particularly our preliminary finding that a doctor’s estimates may be more informative than nurses’ estimates).

## Conclusion

This review has highlighted the wide degree of accuracy reported for the SQ as a prognostic tool. Further work is required to understand the processes by which clinicians arrive at their prognostic estimates, to refine the accuracy of the SQ and to compare its performance against other more sophisticated prognostic tools, particularly in populations where a higher proportion of deaths occur.

## Additional files

Additional file 1: Figure S1. Funnel plot to assess publication bias. (PNG 31 kb)

Additional file 2: Table S1. Studies that were excluded during the full review and the reason for exclusion. (DOCX 18 kb)

Additional file 3: Table S2. Quality rating using the Newcastle–Ottawa Scale. (DOCX 16 kb)
