# Supplementary figures and images for: How accurate is the ‘Surprise Question’ at identifying patients at the end of life? A systematic review and meta-analysis
Source: BMC Med. 2017 Aug 2;15:139. doi: 10.1186/s12916-017-0907-4 (PMC5540432; doi:10.1186/s12916-017-0907-4)

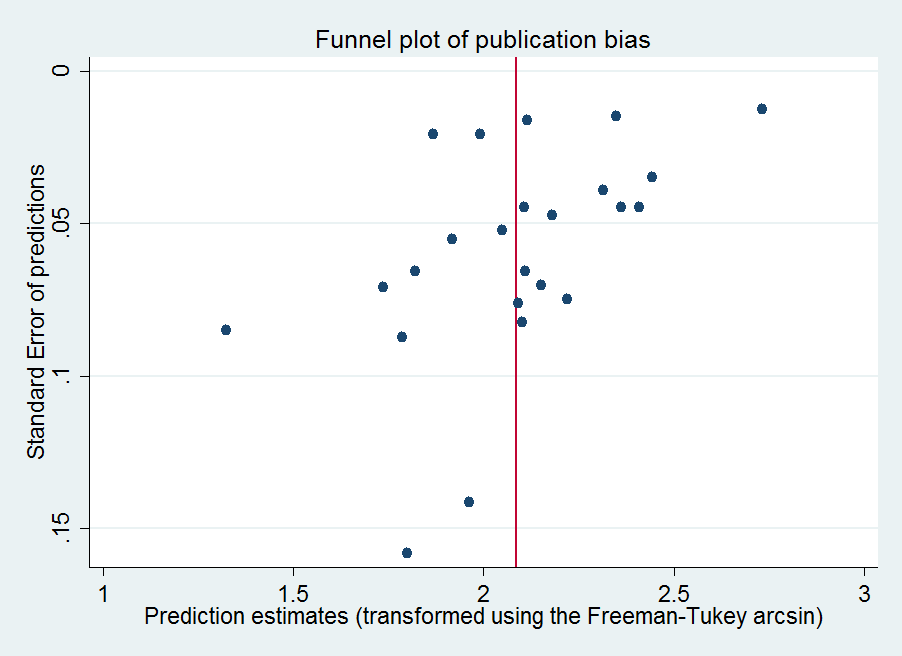

Supplement: Supplementary file 1 — Funnel plot to assess publication bias. (PNG 31 kb) [file 12916_2017_907_MOESM1_ESM.png]
