# Supplementary material for: How accurate is the ‘Surprise Question’ at identifying patients at the end of life? A systematic review and meta-analysis
Source: BMC Med. 2017 Aug 2;15:139. doi: 10.1186/s12916-017-0907-4 (PMC5540432; doi:10.1186/s12916-017-0907-4)
Supplement: Supplementary file 2 — Studies that were excluded during the full review and the reason for exclusion. (DOCX 18 kb) [file 12916_2017_907_MOESM2_ESM.docx]

Additional file 2: Table S1: Studies that were excluded during the full review and the reason for exclusion

| **Study Reference for full review** | **Reason for exclusion** |
| --- | --- |
| Adams, J. A., Dando, N., Barrett, C., Speirs, O., & Clarke, B. J. (2013). Palliative care and stroke: Advanced care planning for stroke patients. [Conference Abstract]. *International Journal of Stroke, 8*, 27. | Retrospective |
| Berkeley, L., Stella, J., Orford, N., Corke, C., Elderkin, T., & Milnes, S. (2014). The appropriateness and adequacy of documented treatment limitations in a tertiary regional Australian hospital: A prospective cohort study. [Conference Abstract]. *EMA - Emergency Medicine Australasia, 26*, 13. | Not about SQ accuracy |
| Bolognesi, D., Moroni, M., Zocchi, D., Abernethy, A., Rondelli, R., Savorani, G., ... & Biasco, G. (2014). Is the “surprise” Question a Useful Trigger to Predict those Advanced Cancer Patients who Could Benefit from Early Palliative Care? A Prospective Study among General Practitioners. Palliative Medicine, 28(6), 565. | Duplicate |
| Briggs, S. R., Vernon, M. J., Yeo, J., Gosling, D., Goodwin, J., & Simcock, S. (2011). Advance care planning in a nursing home service. Can it make a difference? [Conference Abstract]. *Age and Ageing, 40*, ii22. | Retrospective |
| Brothers, H., Gleeson, A., Kilbane, J., Scott, M., Evans, J., Powell, E., & Margetts, S. (2015). Standards of end-of-life care in patients with non-malignant respiratory disease. [Conference Abstract]. *Thorax, 70*, A88. | Retrospective |
| Denvir, M., How Teoh, S., Pender, M., Lane, N., Fenning, S., & Anand, A. (2015). Frailty at discharge is a marker of end-of-life following admission with acute coronary syndrome or acute heart failure. [Conference Abstract]. *Heart, 101*, A26. | Duplicate |
| Gane, M. D., Braun, A., Stott, D., Wellsted, D., & Farrington, K. (2013). How Robust Is the 'Surprise Question' in Predicting Short-Term Mortality Risk in Haemodialysis Patients? [Article]. *Nephron Clinical Practice, 123*(3-4), 185-193. doi: 10.1159/000353735 | Duplicate |
| Gardiner, C., Gott, M., Ingleton, C., Seymour, J., Cobb, M., Noble, B., ... & Ryan, T. (2013). Extent of palliative care need in the acute hospital setting: a survey of two acute hospitals in the UK. Palliative medicine, 27(1), 76-83. | Not about SQ accuracy |
| Gerlach, C., Halbe, L., Hess, G., Wehler, T., Theobald, M., & Weber, M. (2013). «would I be surprised if this patient died in the next year?»-Introducing the «surprise» Question into a university hematology and oncology outpatient clinic. Onkologie, 36, 164-165. | Duplicate |
| Gomez-Batiste, X., Martinez-Munoz, M., Blay, C., Amblas, J., Vila, L., & Costa, X. (2013). Identification of people with chronic advanced diseases and need of palliative care in sociosanitary services: Elaboration of the NECPAL CCOMS-ICO (c) tool. [Article]. *Medicina Clinica, 140*(6), 241-245. doi: 10.1016/j.medcli.2012.06.027 | Not about SQ accuracy |
| Gott, M., Frey, R., Raphael, D., O'Callaghan, A., Robinson, J., & Boyd, M. (2013). Palliative care need and management in the acute hospital setting: a census of one New Zealand Hospital. *Bmc Palliative Care, 12*. | Not about SQ accuracy |
| Gunaratna, N., & Limaye, S. (2014). 51 Recognition of hospitalised patients likely to die within 30 days of hospital discharge - are we missing opportunities to communicate end of life and advance care planning decisions to primary care? *Age & Ageing, 43*(suppl_1), i12-i12. | Retrospective |
| Knestrick, M. W., Pedraza, S. L., Culp, S., Falkenstine, E. C., & Moss, A. H. (2016). Benefits of physician orders for scope of treatment (POST) forms on end-of-life care in cancer patients: Insights from the West Virginia registry. [Conference Abstract]. *Journal of Clinical Oncology. Conference, 34*(no pagination). | Not about SQ accuracy |
| Mason, C. L., & Shah, S. (2012). Identification of patients in the last year of life in the acute hospital setting: Evaluation of the use of modified gold standards framework prognostic criteria. [Conference Abstract]. *Palliative Medicine, 26 (4)*, 469-470. | Not about SQ accuracy |
| Milnes, S., Orford, N. R., Berkeley, L., Lambert, N., Simpson, N., Elderkin, T., . . . Bailey, M. (2015). A prospective observational study of prevalence and outcomes of patients with Gold Standard Framework criteria in a tertiary regional Australian Hospital. [Journal article]. *BMJ supportive & palliative care, 21*, 21. | Not about SQ accuracy |
| Moretti, C., Bertaina, M., Quadri, G., D'Ascenzo, F., Marra, S., Perin, P. C., . . . Gaita, F. (2014). Frailty and end-of-life in acs patients: Identification and prognostic impact. [Conference Abstract]. *Giornale Italiano di Cardiologia, 15*, e28. | Not about SQ accuracy |
| Moretti, C., Fenning, S., Parviz, Y., Gunn, J., D'Ascenzo, F., Giusto, F., . . . Denvir, M. (2013). Frailty assessment as a prognostic tool in elderly acute coronary syndrome patients to identify those approaching end-of-life: Results from prospective multicenter fate-acs study. [Conference Abstract]. *European Heart Journal, 34*, 559-560. | Not about SQ accuracy |
| Moretti, C., Giusto, F., Quadri, G., D'Ascenzo, F., Di Cuia, M., De Simone, V., . . . Gaita, F. (2013). A comparative analysis of cardiovascular and frailty prognostic risk scores in patients with ACS. The STORM study. [Conference Abstract]. *EuroIntervention, 9*, 140. | Not about SQ accuracy |
| Moretti, C., Iqbal, J., Murray, S., Bertaina, M., Parviz, Y., Fenning, S., . . . Denvir, M. (2016). Prospective assessment of a palliative care tool to predict one-year mortality in patients with acute coronary syndrome. [Journal article]. *European Heart Journal Acute Cardiovascular Care, 15*, 15. | Not about SQ accuracy |
| Moretti, C., Quadri, G., D'Ascenzo, F., Bertaina, M., Giusto, F., Marra, S., . . . Gaita, F. (2016). THE STORM (acute coronary Syndrome in paTients end Of life and Risk assesMent) study. [Journal]. *Emergency Medicine Journal, 33*(1), 10-16. | Not about SQ accuracy |
| Moretti, C., Quadri, G., Iqbal, J., Murray, S., Ascenzo, F., Parviz, Y., . . . Denvir, M. (2015). Identifying the last year of life in patients presenting with acute coronary syndrome: A multi-centre prospective study (Fate ACS study). [Conference Abstract]. *EuroIntervention. Conference: EuroPCR*(pagination). | Not about SQ accuracy |
| Moss, A. H., Lunney, J. R., Culp, S., Auber, M., Kurian, S., Rogers, J., ... & Abraham, J. (2010). Prognostic significance of the “surprise” question in cancer patients. Journal of palliative medicine, 13(7), 837-840. | Duplicate |
| Moss, A. H., Lunney, J. R., Culp, S., Auber, M., Kurian, S., Rogers, J., ... & Abraham, J. (2009). Prognostic significance of the “surprise” question in cancer patients. Journal of Clinical Oncology, 27(15S), 9588-9588. | Duplicate |
| Murray, S., & Boyd, K. (2011). Using the ‘surprise question’ can identify people with advanced heart failure and COPD who would benefit from a palliative care approach. *Palliative Medicine, 25*(4), 382-382. doi: 10.1177/0269216311401949 | Review, not data |
| O'Caoimh, R., Gao, Y., Svendrovski, A., Healy, E., O'Connell, E., O'Keeffe, G., . . . Molloy, D. W. (2015). The Risk Instrument for Screening in the Community (RISC): a new instrument for predicting risk of adverse outcomes in community dwelling older adults. [Article]. *Bmc Geriatrics, 15*. doi: 10.1186/s12877-015-0095-z | Not about SQ accuracy |
| Reilly, L., Reilly, K., Mc Closkey, M., Kelly, M., Aziz, A., Carlin, D., ... & Sharkey, R. A. (2013, November). Prognostic Significance of the 'Surprise Question' in an Respiratory Inpatient Population in a DGH. In Irish Journal of Medical Science (Vol. 182, pp. S484-S484) | Retrospective |
| Rhee, J., & Clayton, J. M. (2015). The ‘surprise’question may improve the accuracy of GPs in identifying death in patients with advanced stage IV solid-cell cancer. Evidence Based Medicine, ebmed-2014. | Review, not data |
| Rowlands, M. S., Wilson, J. A., & Thomas, P. K. (2015). Improving end-of-life care in the community. *Primary Care Cardiovascular Journal, 8*(1), 15-17. | Not about SQ accuracy |
| Rudran, B., Idris, L., Childs, C., Riccio, F., Loganathan, S., & Shaw, T. J. (2011). Acute exacerbations of COPD: A review of resuscitation status and associations with prognostic factors in hospital admissions. [Conference Abstract]. *Thorax, 66*, A154-A155. | Not about SQ accuracy |
| Strout, T. D., Haydar, S. A., Han, P. J. K., & Bond, A. G. (2015). 221 Utility of the Modified “Surprise Question” for Predicting Inpatient Mortality in Emergency Department Patients. Annals of Emergency Medicine, 66(4), S81. | Duplicate |
| Szalwinski, B., Lamare, G., Vakil, E., Kousha, M., & Aly, O. (2014). Appropriate Use Of Renal Replacement Therapy In The Medical Intensive Care Unit. In A24. *End Of Life And Stressors In The ICU* (pp. A1137-A1137). *American Thoracic Society.* | Not about SQ accuracy |
| Thomas, K., & Noble, B. (2007). Improving the delivery of palliative care in general practice: An evaluation of the first phase of the Gold Standards Framework. *Palliative Medicine, 21*(1), 49-53. | Not about SQ accuracy |
| Wittenberg, S. M., & Cohen, L. M. (2009). Estimating prognosis in end-stage renal disease. Progress in Palliative Care, 17(4), 165-169. | Review, not data |
