# Supplementary material for: How accurate is the ‘Surprise Question’ at identifying patients at the end of life? A systematic review and meta-analysis
Source: BMC Med. 2017 Aug 2;15:139. doi: 10.1186/s12916-017-0907-4 (PMC5540432; doi:10.1186/s12916-017-0907-4)
Supplement: Supplementary file 3 — Quality rating using the Newcastle–Ottawa Scale. (DOCX 16 kb) [file 12916_2017_907_MOESM3_ESM.docx]

# Additional file 3: Table S2 Quality rating using the Newcastle-Ottowa Scale

|  | Quality Rating Questions | | | | | | | |  |  |  |
| --- | --- | --- | --- | --- | --- | --- | --- | --- | --- | --- | --- |
| First Author | 1 | 2 | 3 | 4 | 5 | 6 | 7 | 8 | Rater 1 | Rater 2 | mean |
| Amro | a) * | a)* | b)* | a) * | a) * | d) | a)* | a) * | 7 | 6 | 6.50 |
| Carmen | b) * | a)* | d) | a) * | none | d) | a)* | a) * | 5 | 5 | 5.00 |
| Cohen | a) * | a)* | a)* | a) * | b) ** | d) | a)* | b) * | 8 | 8 | 8.00 |
| Da Silva Gane | a) * | a)* | b)* | a) * | b) ** | d) | a)* | a)* | 7 | 6 | 6.50 |
| Feyi | a) * | a)* | a)* | a) * | a) * | d) | a)* | a) * | 7 | 5 | 6.00 |
| Gibbins | c) | c) | b)* | a) * | a) * | d) | a)* | b) * | 5 | 5 | 5.00 |
| Halbe | c) | a)* | b)* | a) * | a) * | d) | a)* | b) * | 6 | 5 | 5.50 |
| Hamano | c) | a)* | a)* | a) * | a) * | a)* | a)* | b) * | 7 | 6 | 6.50 |
| Johnson | b) * | a)* | d) | a) * | a) * | d) | a)* | a) * | 6 | 5 | 5.50 |
| Khan | c) | a)* | d) | a) * | none | d) | a)* | a) * | 3 | 3 | 3.00 |
| Moroni | a) * | a)* | a)* | a) * | b) ** | a)* | a)* | a) * | 9 | 8 | 8.50 |
| Moss* | a) * | a)* | a)* | a) * | b) ** | d) | a)* | a) * | 8 | 7 | 7.50 |
| Moss† | a) * | a)* | a)* | a) * | b) ** | d) | a)* | a) * | 8 | 7 | 7.50 |
| O'Callaghan | a) * | a)* | a)* | a) * | a) * | b) * | a)* | a) * | 8 | 6 | 7.00 |
| Pang | a) * | a)* | b)* | a) * | b) ** | d) | a)* | a) * | 8 | 5 | 6.50 |
| Strout | c) | a)* | a)* | a) * | a) * | d) | a)* | a) * | 5 | 4 | 4.50 |
| Thiagarajan | a) * | a)* | a)* | a) * | b) ** | b) * | a)* | a) * | 9 | 6 | 7.50 |
| Lefkowits | c) | c) | a)* | a) * | b) ** | d) | a)* | a) * | 6 | 4 | 5.00 |
| Gopinathan | c) | a)* | d) | a) * | a) * | b) * | a)* | a) * | 6 | 4 | 5.00 |
| South | a) * | a)* | d) | a) * | a) * | d) | a)* | a) * | 6 | 3 | 4.50 |
| Vick | c) | a)* | d) | a) * | none | d) | a)* | a) * | 3 | 3 | 3.00 |
| Fenning | a) * | a)* | a)* | a) * | b) ** | b) * | a)* | a) * | 9 | 9 | 9.00 |
| Haga | b) * | a)* | a)* | a) * | a) * | a)* | a)* | a) * | 8 | 7 | 7.50 |
| Lilley | a) * | a)* | a)* | a) * | b) ** | a)* | a)* | a) * | 9 | 6 | 7.50 |
| Gardiner | a) * | a)* | a)* | a) * | a) * | d) | a)* | a)* | 6 | 5 | 5.50 |
| Barnes | a) * | a)* | d) | a) * | b) ** | d) | a)* | d) | 6 | 3 | 4.50 |

Out of a maximum of 9 points; † 2010; * 2008.
